# Targeting the gut to improve seizure control in CDKL5 deficiency disorder (CDD): study protocol for a single-arm, open-label clinical trial

**DOI:** 10.3389/fneur.2025.1642329

**Published:** 2025-10-28

**Authors:** Francesca Triva, Elisa Borghi, Matteo Domenico Marsiglia, Emerenziana Ottaviano, Emilia Ricci, Paola Tognini, Marco Montecucco, Aglaia Vignoli

**Affiliations:** ^1^Department of Health Sciences, University of Milan, Milan, Italy; ^2^Health Science Interdisciplinary Center, Scuola Superiore Sant'Anna, Pisa, Italy; ^3^Kolfarma s.r.l., Genoa, Italy; ^4^Childhood Neurology and Psychiatry Unit, Azienda Socio Sanitaria Territoriale Grande Ospedale Metropolitano (ASST GOM) Niguarda, Milan, Italy

**Keywords:** CDKL5 deficiency disorder, gut microbiota, seizures, gut-brain axis, sleep disturbance, drug resistant epilepsy, neurodevelopmental disorder

## Abstract

**Introduction:**

Cyclin-dependent kinase-like 5 deficiency disorder (CDD) is a neurodevelopmental condition characterized by infantile-onset epilepsy, developmental delay, intellectual and motor disabilities, sleep disturbances, and cortical visual impairment. Currently, there is no treatment for CDD, and epilepsy is a prominent and severe feature of the disorder. Standard anti-seizure medications have limited efficacy in seizure control, leading to detrimental effects on cognitive and motor development in CDD. The gut-brain axis has gained attention in epilepsy research, prompted by evidence of gastrointestinal (GI) symptoms in people with epilepsy. Notably, CDD patients experience GI problems and exhibit alterations in their gut microbiota compared to healthy individuals. We propose targeting the gut-microbiota-brain axis in CDD patients to alleviate seizures and potentially ameliorate other symptoms.

**Methods and analysis:**

The protocol involves a two-step treatment strategy: a 12-week supplementation with alpha-lactalbumin (ALAC), fructooligosaccharides (FOS), and inulin to reduce inflammation, followed by a 12-week supplementation with ALAC/FOS/Inulin plus Sodium butyrate (NaB), to restore the balance of the gut microbiota. Clinical parameters, including seizure frequency, sleep disturbances, and GI discomfort, will be evaluated. Stool samples will be collected to analyse the gut microbiome. Primary objectives are to determine whether supplementation with ALAC/FOS/inulin alone or in combination with NaB can improve neurological features in CDD and to explore their effects on gut microbiota composition. Our study aims to provide insights into the potential benefits of targeting the gut-brain axis in CDD and offer new therapeutic options to improve seizure control and associated comorbidities.

**Ethics and dissemination:**

The study protocol was approved by the local ethics committee (CET 3, n° 4189_17.04.2024_N_bis). Study results will be disseminated by the investigators through presentations at international scientific conferences and reported in peer-reviewed scientific journals.

**Clinical trial registration:**

ClinicalTrials.gov, Identifier NCT06448663

## Introduction

Cyclin-dependent kinase-like 5 (CDKL5) deficiency disorder (CDD) is a severe neurological condition that primarily affects brain development, leading to significant challenges such as early-onset, treatment-resistant seizures, profound developmental delay, intellectual and motor impairments, sleep issues, and cortical visual dysfunction (1). Most individuals with CDD begin experiencing seizures within the first year of life, with 90% showing symptoms by 3 months of age (2). The disorder is estimated to occur in approximately 1 in 40,000–60,000 live births and predominantly affects females, with a gender ratio of 12:1 (3). CDD is caused by pathogenic variants in the *CDKL5* gene, located on the X chromosome, which encodes a serine/threonine kinase highly expressed in the brain. Given the severe neurological impact of CDD, the *CDKL5* gene plays a crucial role in normal brain development and function, particularly in synaptic plasticity and neuronal communication (4). Individuals with CDD often experience various types of seizures, including epileptic spasms (with or without hypsarrhythmia), tonic seizures, generalized tonic–clonic seizures, and complex seizures with multiple phases (such as the hypermotor-tonic-spasms sequence) (5). Epilepsy in CDD patients is highly refractory, and there are few reports concerning response to standard treatment regimens. Alternate therapies are not yet established. Ketogenic diet and vagus nerve stimulation showed heterogeneous results in observational studies (6).

While seizures are a prominent and early symptom of CDD, other neurological issues such as global hypotonia, sleep disturbances, behavioral challenges, movement disorders, and difficulties with swallowing are also significant (7).

Emerging evidence indicates the crucial role of gut microbiota in neurodevelopment through a complex interplay of immune, neuronal, and systemic endocrine pathways. The microbiota–gut–brain axis (MGBA) has been implicated in neurodevelopmental disorders, including autism spectrum disorder, attention deficit hyperactivity disorder, and Rett Syndrome (8).

Recently, the MGBA has been investigated for its involvement in epilepsy (9), starting from the evidence that people with epilepsy (PWE) often show GI symptoms, and patients with inflammatory bowel disease have a higher susceptibility to epilepsy (10). Several studies have suggested significant changes in the fecal microbial composition in animal models of seizures and between PWE and healthy subjects (11–15). Notably, CDD patients experience GI issues, and recent data suggest that gut microbial composition differs from healthy relatives, showing a more pro-inflammatory profile (16). Alterations in the gut microbiota composition might affect microbial metabolites production and release, which in turn can modulate gut homeostasis, local and systemic inflammation (17).

CDD symptoms severely affect patients’ quality of life, and no approved treatments currently exist. Disease management primarily focuses on symptom relief, particularly seizure control, through medications, alternative therapies, and supportive care (18).

The modulation of gut microbiota could potentially alleviate some neurological symptoms by reducing inflammation and improving gut-brain communication (19, 20).

To investigate this strategy, we designed a 32-week interventional study to evaluate possible beneficial effects of special purpose foods in improving CDD clinical phenotype. The protocol’s dietary supplements contain alpha-lactalbumin (ALAC), inulin, fructooligosaccharides (FOS), with/without sodium butyrate (NaB). ALAC is a whey protein that is abundant in human milk, rich in essential amino acids, particularly tryptophan, which is a precursor for serotonin, a neurotransmitter that plays a crucial role in mood regulation, cognitive function and central nervous system homeostasis (21). The balance between pro-inflammatory and anti-inflammatory metabolites generated from tryptophan metabolism is crucial; while some metabolites like kynurenic acid are neuroprotective, others, such as quinolinic acid, can be neurotoxic (22). FOS and inulin are well-known prebiotics that promote the growth and metabolic activity of beneficial intestinal bacterial genera, particularly *Bifidobacterium* and *Lactobacillus*, enhancing SCFA production and supporting gut health (23, 24).

Butyrate, on the contrary, a postbiotic, can exert anti-inflammatory effects, ameliorate oxidative stress-induced damage on intestinal cells, promoting a healthier microbial profile that can lead to improved gastrointestinal function (25, 26).

## Methods and analysis

### Trial objectives

The main objective of the current protocol is to investigate whether ALAC/FOS/Inulin supplementation itself or plus NaB can represent a valid option to reduce seizure frequency in CDD patients.

Moreover, since associated comorbidities have a remarkable impact on patients and families, the goal of improving these symptoms represents a worthy endeavor while awaiting a more definitive cure.

### Trial design

**Figure 1:**
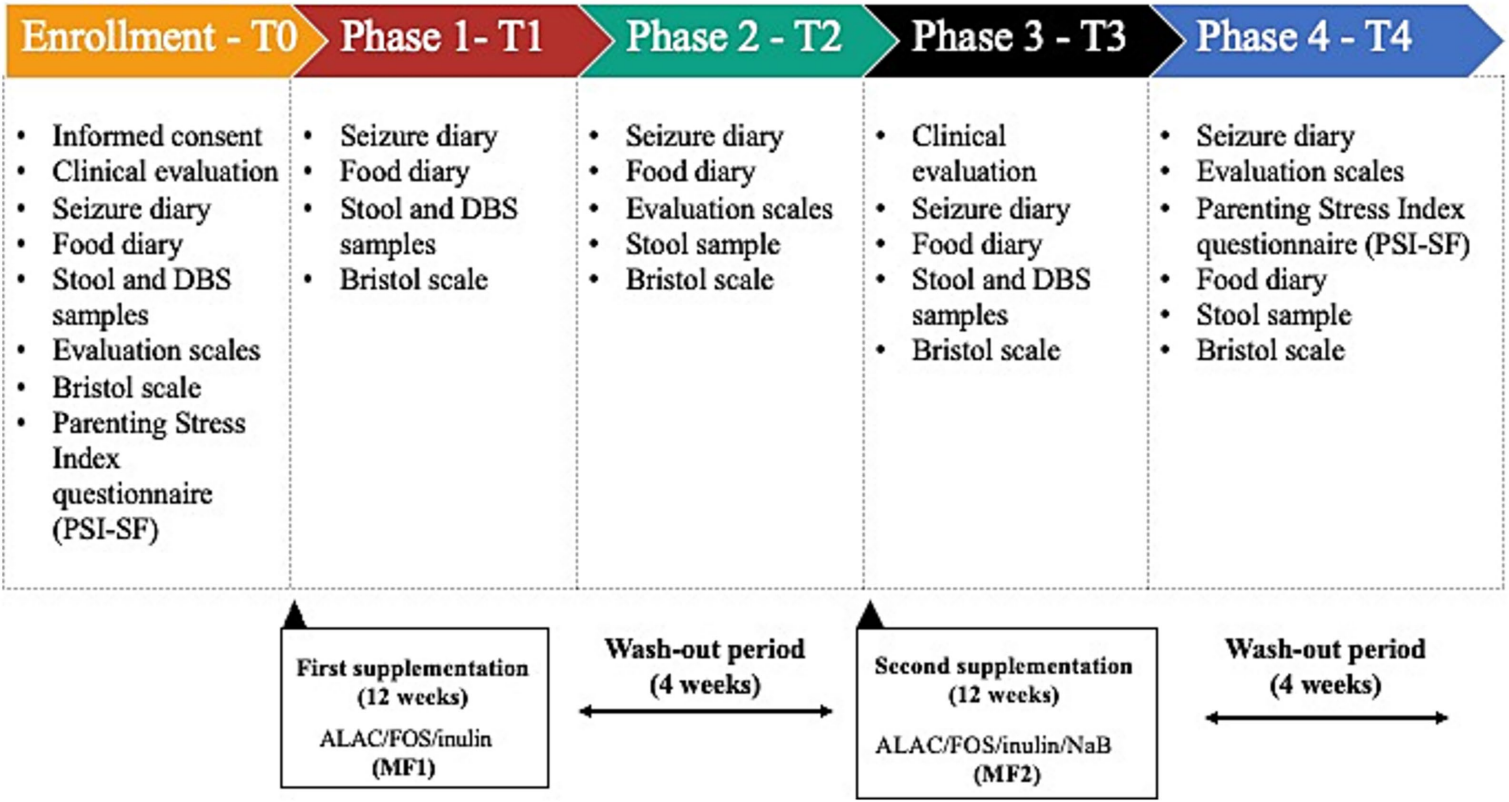
Trial design. We planned a 32-week longitudinal intervention based on two sequential interventions. A 12-weeks supplementation has been selected to ensure a sufficient duration for evaluating meaningful clinical changes (according to the primary and secondary outcomes). Wash out periods have been designed to verify the duration of clinical and gut microbiota changes after supplement discontinuation. DBS, dried-blood spot; ALAC, alpha-lactalbumin; FOS, Fructooligosaccharides; MF, Medical Food; NaB, Sodium butyrate.

This is a 32-week single-arm, open-label clinical trial of ALAC/FOS/Inulin (MF1, Kolfarma s.r.l. - Italy) vs. ALAC/FOS/Inulin/NaB (MF2, Kolfarma s.r.l. - Italy) aimed at restoring gut homeostasis and gut community health (Figure 1). The intervention will be conducted in a single investigational study centre in Italy, namely the Child and Adolescent Childhood Neurology and Psychiatry Unit at ASST Grande Ospedale Metropolitano Niguarda in Milan, Italy.

We planned an interventional study as follows: a 12-week supplementation period with MF1, 4-week washout, 12-week supplementation with MF2, and a final 4-week washout. Patients will undergo full clinical assessments at baseline (enrollment, before starting the first supplementation), after the first supplementation and at the end-of-treatment visit, 1 week after the last supplementation. Stool and dried-blood spot (DBS) samples, food diary, and GI scales will be collected at scheduled time points, as described in the Study Procedures’ paragraph. Gut microbiome characterization, SCFAs (short-chain fatty acids) quantification, clinical scales and dietary intake will be assessed.

### Study treatment

The study involves the use of two medical foods (MF1 and MF2 - Kolfarma s.r.l.) containing ALAC, inulin, FOS, with the addition of NaB in a CDD patient population. Both products are indicated for pediatric age (> 3 years). The product must be taken orally and not parenterally.

MF1 is a dietary supplement aimed at improving intestinal dysbiosis containing ALAC (contains milk derivatives), FOS, inulin, sweetener (steviol glycosides from stevia), emulsifier (polysorbate 80).

- The first-round supplementation will be administered for 12 weeks. One dose/day (2 g sachet) is intended to be administered orally once a day after dissolving in water.

MF2 is a special medical purpose food intended for the dietary treatment of drug-resistant epilepsy. It contains ALAC (contains milk derivatives), inulin, FOS, flavors, sucralose, polysorbate 80, and NaB.

- The second-round supplementation will be administered for 12 weeks. For participants weighing <30 kg, a 4 g dose (i.e., one 4 g sachet) is intended to be administered orally once a day after dissolving in water, 15 min before meal. For participants weighing ≥30 kg, a 4 g dose (i.e., 4 g sachets) is intended to be administered orally twice a day (12 h interval) after dissolving in water, 15 min before meals.

Intake is not recommended in cases of proven hypersensitivity to one or more ingredients.

No known side effects are reported for the two products used in this study.

If a treatment tolerance problem should develop at any time during the study, the investigator may reduce the patient’s dose, with administration on alternate days. Patients unable to tolerate the treatment even after a dose interruption should be suspended from the study.

### Prohibited therapies

To reduce the effect of spurious variables on study endpoints, therapeutic stability is expected from at least 4 weeks prior to enrolment. The use of antibiotics is not recommended during the study, as it may alter the gut microbiota. However, if deemed necessary, a doctor may prescribe them, provided that the research team is informed. There are no contraindications to taking other drugs during the study.

Any change in treatment will be recorded in the medical record.

### Participants

The study will enrol 20 CDD patients. The sample size for this study is dictated by the rarity of the condition, 1:40,000 live births (3), and considering that the number of CDD patients in Italy is about 60 (according to the report of the Patients Association “CDKL5 Insieme verso la cura”). The sample size is calculated based on the following assumptions: (1) the clinical success for an enrolled patient is defined as a reduction of at least 50% of the seizure frequency compared to baseline; (2) a clinically significant result is achieved if at least 35% of the enrolled patients reduce the seizure frequency by at least 50%.

With these premises, a McNemar exact test (“after vs. before”), applied to a sample of 20 subjects, reaches a power of 80%, less than an alpha-error of 5%, in verifying clinical success in 35% of cases, using the following inputs: percentage of positives that become negative = 0; percentage of negatives that become positive = 35.

Eligible patients from Italy who meet the defined inclusion and exclusion criteria will be recruited, after signing the informed consent.

#### Study inclusion criteria

- Documented genetic diagnosis of CDD based on pathogenic variants in the *CDKL5* gene;
- age range 3–50 years; clinical diagnosis of CDD and demonstrated *CDKL5* pathogenic variant;
- drug-resistant seizures;
- stable drug regimen for 4 weeks prior to starting the study;
- written informed consent signed by the parent/legal guardian/representative prior to the screening visit;
- the caregiver must be able to understand the instructions and consciously participate in the study.

#### Study exclusion criteria

- Enrolment in another clinical trial
- organic GI disorders (i.e., food allergies, celiac disease)
- special diets
- use of percutaneous endoscopic gastrostomy tube (PEG)
- use of antibiotics or probiotics in the month prior to enrolment

### Study procedures

The schedule of activities planned during the study is summarized in Table 1.

**Table 1:** Summary of measures and instruments used in the present study.

Study period					
Enrolment		Intervention period			
Timepoint	T0	T1	T2	T3	T4
Enrolment:					
Eligibility screen	X				
Informed consent	X				
Demographics	X				
Medical history	X				
Concomitant medication	X				
Intervention:					
ALAC, FOS, and inulin supplementation		X			
ALAC, FOS, inulin and NaB supplementation				X	
Assessments					
Dietary survey	X	X	X	X	X
Gastrointestinal Severity Index (GISI) (16) scale	X		X		X
Bristol Stool Form Scale (BSFS) (16)	X	X	X	X	X
Clinical global impression– improvement scale (CGI-I) (16)	X		X		X
Motor-Behavioral Assessment Scale (MBAS) (16)	X		X		X
CDKL5 severity assessment (CDD-SA) (29)	X		X		X
Sleep Disturbance Scale for Children (SDSC) (16)	X		X		X
Daily Seizure diary	X	X	X	X	X
Parenting Stress Index questionnaire (PSI-SF) (16)	X				x
Feces collection	X	X	X	X	X
Dried blood spots collection	X	X		X	
Adverse effects	X	X	X	X	X

T0, screening/baseline; T1, first supplementation (MF1); T2, wash-out period; T3, second supplementation (MF2); T4: wash-out period.

### Subject withdrawal

Patient parents/legal guardians may decide to terminate the study at any time.

The principal investigator, guarantor of the study, may decide to terminate a patient’s participation at any time if the enrolled subject presents adverse events or is not compliant with the study procedures, or after withdrawal of participation consent.

### Data management

Case report forms (CRFs) will be completed for each enrolled study subject, with data consistently recorded on electronic CRFs in alignment with source documents. The data will be securely stored in a centralized data repository (Network Attached Storage) to ensure protection. Each subject will be assigned a unique ID for accurate tracking and management.

### Efficacy endpoints

The primary outcomes of the study are:

- To evaluate the number of CDD patients considered treatment responders (seizure reduction ≥50%, ≥75% or ≥100% from baseline in monthly seizure counts) during the 12-week treatment period in 1st and 2nd round of supplementation. With regard to seizure counting, in order to improve its reliability, seizures characterized by a motor component of sufficient intensity and/or duration to potentially cause a fall if the patient were standing will be analyzed separately. Accordingly, the analysis will focus on epileptic spasms, myoclonic seizures, and tonic seizures. Moreover, to ensure greater oversight of the reliability, caregivers will undergo a brief training on the correct classification and quantification of seizures. Specifically, the type of seizure experienced by the patient will be defined in collaboration with the physician, also using home videos and video-electroencephalographic recordings (videoEEGs) before starting the seizure diary.
- To identify indicator species—specific microbial taxa that are strongly associated with distinct microbial environments—which may serve as biomarkers to guide clinicians in selecting interventions with the highest likelihood of improving patient quality of life.

Secondary outcomes of the study are related to sleep disturbances and GI discomfort. Thus, we will consider:

- Decreased Sleep SDSC scale (max score = 125) by at least 5%;
- Decrease GISI (max score = 17), by at least 2 points.

Both gastrointestinal and sleep disturbances are reported in over 80% of patients with CDKL5 deficiency disorder; therefore, we considered these changes to be clinically meaningful, even if modest in extent, also based on our clinical experience (15).

Other outcomes to be evaluated:

- Global change from baseline, assessed by the Clinical Global Impression of Change (CGI-C), [ranging from 1 (very much improved) to 7 (very much worse), with a score of 4 indicating no change] decrease of at least 1 point;
- Caregiver burden by Parenting Stress Index (PSI-SF); clinically significance > 85%,
- Decrease by at least 5%.

### Biomarker endpoints

The biomarkers evaluation will enhance our understanding of drug safety and its potential effects on both the microbiota and the brain. This investigation will assess the baseline composition of the microbiota and its changes following treatment by examining alpha and beta diversity, the relative abundances of bacterial and fungal taxa, and levels of SCFAs in fecal and DBS samples. Furthermore, clinical evaluations will provide a comprehensive view of patient health by considering disease severity, overall well-being and gastrointestinal improvements over time.

### Statistical analyses

We will examine longitudinal changes, with particular emphasis on differences between baseline (T0) and post-MF1 (T1), as well as between the second baseline following washout (T2) and post-MF2 (T3).

In descriptive analyses, data will be presented as mean ±SD for continuous variables and as percentages for categorical variables. The primary endpoint will be measured using the McNemar exact test. Differences in continuous variables (scale scores, biomarkers) will be tested using the unpaired and paired t-test for independent and dependent samples, respectively, in case of normally distributed data. For non-normally distributed data, the Wilcoxon rank-sum test will be used for independent samples, and the Wilcoxon signed-rank test for dependent samples. For comparisons among multiple time points, the ANOVA (or Kruskal-Wallis test for non-parametric data) will be applied to independent samples, while repeated measures ANOVA (or Friedman test for non-parametric data) will be used for dependent samples. When appropriate, a *post hoc* test (e.g., Bonferroni correction or Tukey’s HSD) will be performed to adjust the *p*-value.

A p-value < 0.05 will be considered statistically significant.

### Registration and guidelines

The study protocol reported here was written in compliance with the Standard Protocol Items, Recommendations for Interventional Trials (27).

The sponsor (no-profit) is Telethon Foundation, Seed grant CDKL5 Renewal 2023 caLL.

Kolfarma s.r.l. holds the license for the study supplements and is responsible for its supply. It has contributed to defining the eligibility criteria and treatment schedule for the study.

### Written informed consent

Participant parents or caregivers will receive an information sheet about the study along with an informed consent form for the collection of biological material. They will also be provided with a telephone number and an email address for any inquiries or to communicate their decision to withdraw from the study at any time.

### Patient confidentiality

To protect patient privacy, all CRFs, banked study samples, study drug accountability records, study reports, and communications will identify patients only by their assigned identification number. Patient confidentiality will be maintained and will not be publicly disclosed, except as permitted by applicable laws and regulations.

### Patient and public involvement

Patients and the public were not involved in developing the study protocol; however, the Associazione CDKL5 “Insieme Verso la Cura” supported subject enrollment and contributed to funding allocation.

## Discussion

CDD is a severe neurodevelopmental condition, characterized by early-onset seizures, intellectual disability, and motor impairments due to pathogenic variants in the *CDKL5* gene. The management of CDD is complex, particularly due to the associated treatment-resistant epilepsy and multiple co-occurring conditions.

Recently, the gut microbiota has emerged in influencing neurological outcomes (6, 20). CDD microbiota has been suggested to be enriched in specific bacterial taxa that seem to correlate with symptoms severity, such as *Lachnoclostridium* and *Enterobacteriaceae* with severe GI symptoms and *Peptostreptococcaceae, Coriobacteriaceae (Collinesella),* and *Erysipelotrichaceae* with daily epileptic seizures (16).

Emerging evidence suggests that modulation of the MGBA through approaches such as prebiotics, probiotics, postbiotics, and fecal microbiota transplantation (FMT) may play a promising role in the management of neurological disorders, including epilepsy^8.^

Thus, *ad hoc* medical foods and/or dietary modifications and/or probiotics may be considered as complementary treatment for CDD patients. These interventions may help restore gut microbiota balance, which has been linked to neurological health (28). The formulation MF2 has been investigated for a clinical trial study for the treatment of Rett syndrome (Trial registration number - ClinicalTrials.gov Identifier NCT05420805), a neurodevelopmental disorder that shares several clinical features with CDD (28). MF2 (ALAC/FOS/inulin/NaB) could offer a synergistic approach to managing CDD by improving gut health and reducing inflammation and may help to address both the gastrointestinal and neurological symptoms experienced by patients.

In conclusion, CDD is a debilitating and life-threatening neurodevelopmental disorder for which no therapies are available that address its core features. The study protocol presented here provides a potentially viable treatment for the core signs and symptoms of CDD, ranging from seizures to GI and sleep problems, and supports further trials in this population. The results of this study could pave the way for new therapeutic opportunities. Our aim is to significantly enhance the quality of life for patients and their caregivers, recognizing the pressing needs of individuals and families affected by this condition.
